# Whole genome sequencing of *Plasmodium falciparum* from dried blood spots using selective whole genome amplification

**DOI:** 10.1186/s12936-016-1641-7

**Published:** 2016-12-20

**Authors:** Samuel O. Oyola, Cristina V. Ariani, William L. Hamilton, Mihir Kekre, Lucas N. Amenga-Etego, Anita Ghansah, Gavin G. Rutledge, Seth Redmond, Magnus Manske, Dushyanth Jyothi, Chris G. Jacob, Thomas D. Otto, Kirk Rockett, Chris I. Newbold, Matthew Berriman, Dominic P. Kwiatkowski

**Affiliations:** 1 Wellcome Trust Sanger Institute, Hinxton, CB10 1SA UK; 2 MRC Centre for Genomics and Global Health, University of Oxford, Oxford, OX3 7BN UK; 3 Wellcome Trust Centre for Human Genetics, University of Oxford, Oxford, OX3 7BN UK; 4 Weatherall Institute of Molecular Medicine, University of Oxford, Oxford, OX3 9DS UK; 5 International Livestock Research Institute, Box 30709, Nairobi, Kenya; 6 Navrongo Health Research Centre, Post Office Box 114, Navrongo, Ghana; 7 Noguchi Memorial Institute for Medical Research, University of Ghana, P. O. Box LG 581, Legon, Accra, Ghana; 8 Broad Institute, 415 Main St, Cambridge, MA 02142 USA; 9 Addenbrooke’s Hospital, University of Cambridge School of Clinical Medicine, Hills Rd, Cambridge, CB2 0SP UK

**Keywords:** Malaria, Dried blood spot, Selective whole genome amplification, Field samples, Whole genome sequencing

## Abstract

**Background:**

Translating genomic technologies into healthcare applications for the malaria parasite *Plasmodium falciparum* has been limited by the technical and logistical difficulties of obtaining high quality clinical samples from the field. Sampling by dried blood spot (DBS) finger-pricks can be performed safely and efficiently with minimal resource and storage requirements compared with venous blood (VB). Here, the use of selective whole genome amplification (sWGA) to sequence the *P. falciparum* genome from clinical DBS samples was evaluated, and the results compared with current methods that use leucodepleted VB.

**Methods:**

Parasite DNA with high (>95%) human DNA contamination was selectively amplified by Phi29 polymerase using short oligonucleotide probes of 8–12 mers as primers. These primers were selected on the basis of their differential frequency of binding the desired (*P. falciparum* DNA) and contaminating (human) genomes.

**Results:**

Using sWGA method, clinical samples from 156 malaria patients, including 120 paired samples for head-to-head comparison of DBS and leucodepleted VB were sequenced. Greater than 18-fold enrichment of *P. falciparum* DNA was achieved from DBS extracts. The parasitaemia threshold to achieve >5× coverage for 50% of the genome was 0.03% (40 parasites per 200 white blood cells). Over 99% SNP concordance between VB and DBS samples was achieved after excluding missing calls.

**Conclusion:**

The sWGA methods described here provide a reliable and scalable way of generating *P. falciparum* genome sequence data from DBS samples. The current data indicate that it will be possible to get good quality sequence on most if not all drug resistance loci from the majority of symptomatic malaria patients. This technique overcomes a major limiting factor in *P. falciparum* genome sequencing from field samples, and paves the way for large-scale epidemiological applications.

**Electronic supplementary material:**

The online version of this article (doi:10.1186/s12936-016-1641-7) contains supplementary material, which is available to authorized users.

## Background

The last decade has seen rapid advances in whole genome sequencing technologies helping to track disease outbreaks and the spread of drug resistance genes [1]. Clinical and public health applications for *Plasmodium falciparum* sequencing rely on obtaining sequenceable material from samples collected in the field, often in resource-limited conditions. To date, the practical difficulties in sample collection, storage and transportation impose significant barriers to the use of genomic approaches for malaria surveillance.

The most practical and convenient method for sampling clinical malaria parasites is through small blood volumes obtained from capillary blood using finger or heel-pricks [2, 3]. These small blood samples—about 50 µl in volume—are blotted on filter papers for efficient transportation and storage without requiring refrigeration; this is especially applicable to resource-deprived regions where the disease is endemic. Despite the convenience and ease of sampling, DNA extracted from dried blood spot (DBS) filter papers often has low parasite DNA yield and an overwhelming host DNA contamination, which poses serious limitations in downstream genetic analyses [4]. These technical bottlenecks have prevented analysis of large numbers of pathogen samples collected by DBS at whole genome resolution, including archived clinical specimens, using current high throughput sequencing technologies.

Currently, whole blood from malaria patients used for *P. falciparum* sequencing is obtained through venous blood (VB) draws. This requires skilled phlebotomists or clinicians with appropriate training. Once collected, VB samples are processed by filtering out leucocytes using cellulose columns [5] and require refrigerated storage followed by centrifugation and blood pellet freezing or DNA extraction. These requirements limit the scope for sample collection in remote regions where healthcare infrastructure is already under strain. The cellulose filtration process, although very effective in parasite enrichment, requires large volumes of blood (>2 ml) [6]. Such volumes can be difficult to obtain, especially from young children, who may already be anaemic as a result of *P. falciparum* infection [7] and who bear the heaviest disease burden globally.

To overcome the challenges of low sample quality and quantity, and to allow timely genetic analysis of clinical samples collected directly from patients without culture adaptation, an approach was used that selectively amplifies parasite DNA from low blood volume clinical samples. The selective whole genome amplification (sWGA) strategy, originally described by Leichty and Brisson [8], uses computationally selected short oligonucleotide probes of 8–12 mers as primers that preferentially bind to the target genome, and this approach has been successfully applied to *Laverania* parasites, including *P. falciparum* [9, 10]. The purpose of the present study was to undertake a detailed evaluation of sWGA approaches for sequencing the *P. falciparum* genome from dried blood spots.

## Methods

### Primer design and selection

**Fig. 1:**
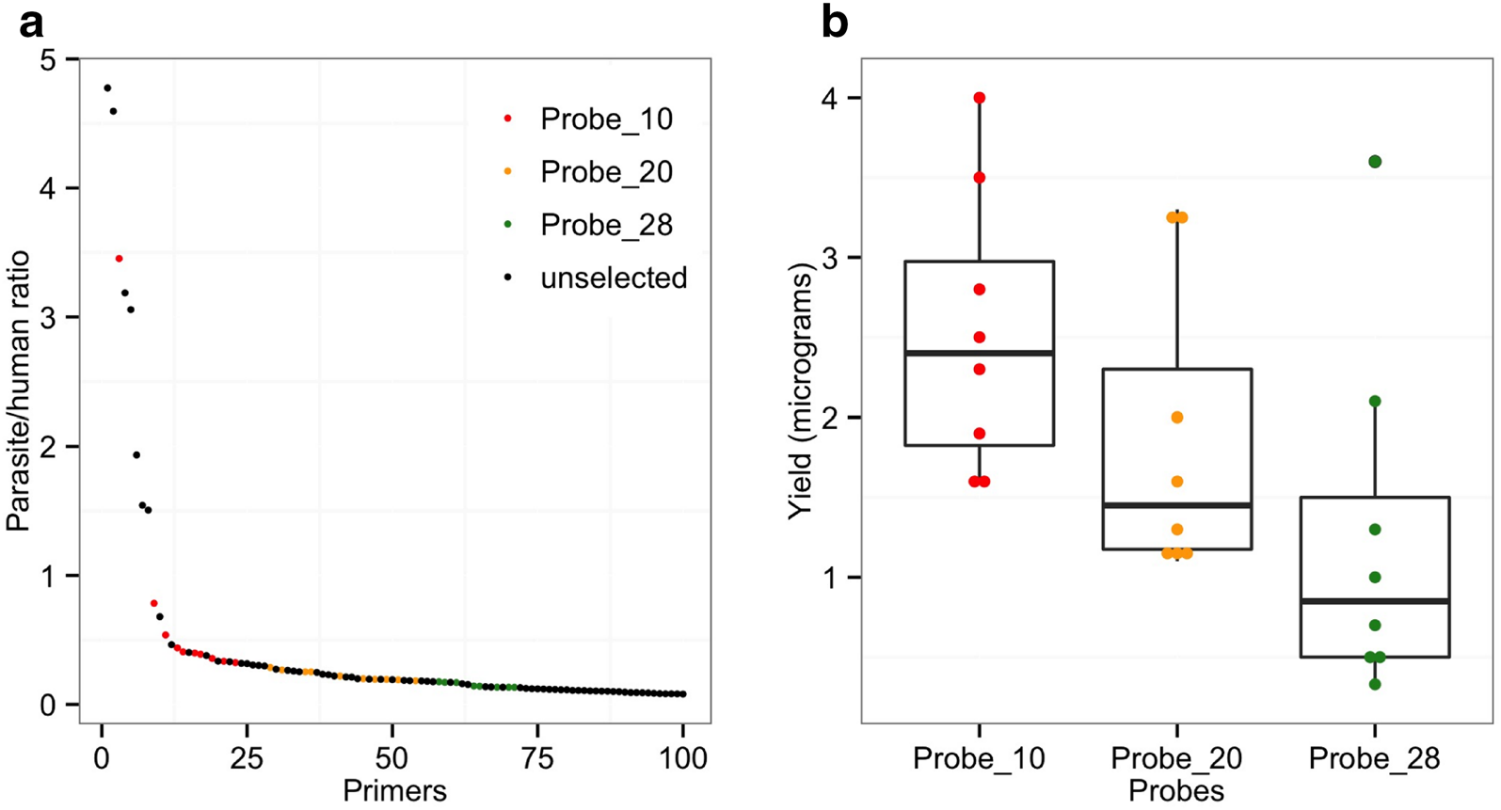
Primer selection analysis. **a** The frequency of each primer as ranked by the frequency of occurrence. The y-axis represents the calculated ratio of the frequency in the parasite (desired) genome against frequency in the human (contaminating) genome. The x-axis shows the order of ranking by the frequency of occurrence ratio. **b** DNA yield obtained following the selective whole genome amplification with different pools of probe sets. Probe_10 represent a pool of the first top 10 ranking primers. Probe_20 is a cumulative mixture consisting of all the Probe_10 primers plus the next 10 probes in that order. Probe_28 is a cumulative mixture of all the first 28 primers (Probe_10, Probe_20 and the next 8 probes in the order of frequency ranking)

In order to design probes that preferentially bind to the *P. falciparum* genome, a published PERL script [8] was used to select up to 100 (8–12 mer) primers with a predicted specified melting temperature (≤30 °C). The frequency of these primer sequences in the desired (D) *Plasmodium falciparum* 3D7 genome were compared to the contaminating (C) human genome (Fig. 1a). Top 50 primers with the highest desired/contaminating (D/C) ratios were selected for further analysis. From these 50, primers with more than three complementary nucleotides at 3′ and 5′ ends were removed to prevent formation of hairpin structures. To prevent primer–primer dimerization, primer pairs with more than three complementary nucleotides at their ends were also removed. A final 28 primers that passed the above quality control were ordered from Integrated DNA Technologies (Coralville, IA) as standard desalting purification with a single modification of phosphorothioate bond between the last two 3′ nucleotides to prevent primer degradation by the Phi29 polymerase exonuclease activity. Individual primers were reconstituted in Tris HCl (pH 8.0) buffer and pooled into three sets (probes) following the D/C ranking described above: the first set consisted of the first 10 primers (Probe_10), the second set consisted of the first 20 primers (Probe_20), and the third set consisted of all 28 primers (Probe_28). Each set was evaluated to determine which should be taken forward for further assessment.

### Mock samples to test the efficacy of sWGA

To test whether selected primers would successfully amplify the parasite genome, mock clinical samples were prepared by mixing culture-infected red blood cells (infected with *P. falciparum* strain 3D7) with uninfected human whole blood to obtain a simulated parasitaemia ranging from 0.0001 to 1%. *P. falciparum* strain 3D7 parasites for the mock samples were cultured in human O+ erythrocytes with heat-inactivated 10% pooled human serum, as described in [11]. All parasitaemia calculations were based on the estimation of approximately 4 million red blood cells per microlitre of whole blood. DNA was extracted from the samples (N = 8) without leucodepletion. In addition, 6 other mock DNA samples were manually reconstituted by mixing *P. falciparum* genomic DNA with host (human) genomic DNA to obtain parasite/host DNA mixtures of the ratio 1: 24 (4% parasite and 96% human DNA) that were used to investigate genome coverage following sWGA.

### Whole genomes from dried blood spots

To test the efficacy of sWGA in generating reliable genomic data from DBS, WGS datasets obtained from standard leucodepleted-genomic DNA (gDNA) were compared with sWGA-DBS samples. The gDNA samples were derived from two to three ml of venous blood which were then processed by CF-11 or MN filtration to remove leucocytes [5, 6] prior to DNA extraction. On the other hand, the DBS samples were collected by spotting (on filter paper) 50 μl of whole blood obtained by finger pricking. In total, samples from 156 patients that were positive for *P. falciparum* clinical malaria based on rapid diagnostic test (RDT) with CareStart™ Malaria kit (Access Bio Inc, USA) were analysed. Eighty-four DBS were collected from the Kassena-Nankana Districts of Upper East Ghana, of which 48 had matching VB pairs; and 72 DBS were collected from Noguchi Memorial Hospital in Accra, Ghana, all of which had matching VB samples.

### DNA extraction and quantification

Two to three ml of VB samples (mock blood or field) were used to extract DNA, using QiAamp DNA blood midi kit (Qiagen) following the kit manufacturer’s instructions. For DBS, DNA was extracted using QIAamp DNA Investigator Kit (Qiagen, Valencia, California, United States). Approximately 1.5 cm (0.6 in) diameter DBS circles from each filter paper were cut out into small pieces of 3 mm diameter using a single-hole paper punch. Punched pieces from each sample were placed into 2 ml micro-centrifuge tubes from which DNA was extracted following the manufacturer’s instructions except for the reagent volumes and incubation times, which were doubled to accommodate the increased amount of DBS used per sample. An average of 116 ng (standard deviation, SD, 116.7) of DNA was obtained from the DBS extracts out of which at least 5 ng was used as template for sWGA amplification reaction.

### Selective whole genome amplification (sWGA)

The sWGA reaction was performed in 0.2 ml PCR-tubes or plates. The reaction (50 µl total volume) containing at least 5 ng of template DNA, 1× BSA (New England Biolabs), 1 mM dNTPs (New England Biolabs), 2.5 µM of each amplification primer, 1× Phi29 reaction buffer (New England Biolabs), and 30 units of Phi29 polymerase (New England Biolabs), was placed in a PCR machine (MJ thermal Cycler, Bio-Rad) programmed to run a “stepdown” protocol consisting of 35 °C for 5 min, 34 °C for 10 min, 33 °C for 15 min, 32 °C for 20 min, 31 °C for 30 min, 30 °C for 16 h then heating at 65 °C for 15 min to inactivate the enzymes prior to cooling to 4 °C. Once the product was amplified, it was quantified using Qubit^®^ dsDNA high sensitivity (Thermo Fisher Scientific) to determine whether there was enough material for sequencing—minimum required is 500 ng of product. Standard whole genome amplified (WGA) products of the test samples were also sequenced as control to determine the extent of enrichment [12].

### Library preparation of amplified samples and short read high throughput sequencing

sWGA products (≥500 ng total DNA) were cleaned using Agencourt Ampure XP beads (Beckman Coulter) following manufacturer’s instructions. Briefly, 1.8 volumes of beads per 1 volume of sample were mixed and incubated for 5 min at room temperature. After incubation, the tube containing bead/DNA mixture was placed on a magnetic rack to capture the DNA-bound beads while the unbound solution was discarded. Beads were washed twice with 200 µl of 80% ethanol and the bound DNA eluted with 60 µl of EB buffer. Cleaned amplified DNA products (~05–1 µg DNA) were used to prepare a PCR-free Illumina library using the NEBNext DNA sample preparation kit (New England Biolabs) for high throughput sequencing. DNA libraries were sequenced at the Wellcome Trust Sanger Institute using Illumina HiSeq 2500 instruments and Illumina V.3 chemistry. Paired-end sequencing was performed with 100-base reads and an 8-base index read. 12-multiplex sample libraries were loaded to target at least 20 million reads per sample.

### Data analysis

Sequence data obtained from each sample was subjected to standard Illumina QC procedures and 20 million reads per sample was subjected to detailed analysis for enrichment, quality, content, and coverage. Each dataset was analysed independently by mapping sequence reads to the 3D7 reference genome using BWA [13]. SAMtools [14] was used to generate coverage statistics from the BWA mapping output. For enrichment analysis, the number of reads mapping to either host, or *P. falciparum* reference sequences was counted. For genotype and concordance analysis, variant calls were generated using SAMtools mpileup (V0.1.1.19; with the following parameters: -DSV -C50 -m2 -F0.0005 -d 10,000 -gu) and bcftools (V0.1.17; with the following parameters: -p 0.99 -vcgN). A list of 1,241,840 (1.2 million) high-quality single-nucleotide polymorphism (SNP) positions, which were not filtered by gene class or region, but on individual properties of SNPs (such as uniqueness of the surrounding region and within an exon) [15, 16] was used. In silico genotyping of both the DBS (sWGA) and VB (leucodepleted and unamplified) samples was performed using mpileup to count alleles present in at least five reads (alleles with less than five reads were discarded). Although *P. falciparum* is haploid, it is common to find heterozygous calls due to the presence of multiple clonal infections in the same host. In order to genotype heterozygous sites, the 5/2 rule was applied, which requires at least two reads in both reference and alternative alleles, and the sum of both has to be higher than five reads [15]. SNP call concordance analysis between matching DBS and VB samples was performed on sequenced data targeting SNPS present in the core genome as well as key malaria drug resistance genes, such as *crt* (K76T involved in chloroquine resistance) [17], *dhfr* (N51I, involved in pyrimethamine resistance) [18], *dhps* (A581G, involved in sulfadoxine resistance) [19], *mdr1* (N86Y, involved in multiple drugs including mefloquine) [20], and *kelch13* (C580Y, involved in artemisinin resistance) [1].

## Results

### sWGA primer selection and amplification yield

Selected 28 primers were analysed individually (Fig. 1a) to determine their expected binding sites and distribution pattern across the *P. falciparum* genome. Each 1 or 2 kb block had at least one primer binding (Additional file 1: Figure S1). These 28 primers were pooled into three different sets (probes): Probe_10 (consisting of the first 10 primers), Probe_20 (consisting of the first 20 primers), and Probe_28 (a pool of all the 28 primers). In separate reactions, the three probes were used to amplify 5 ng of simulated mock samples (a mix of 3D7-infected red blood cells with uninfected human whole blood; N = 8) to determine which set gives optimal genome amplification and coverage. The amplified products were cleaned and the DNA quantified using Quant-iT™ PicoGreen^®^ dsDNA assay kit (Invitrogen) to determine the yield for each primer pool (Fig. 1b). Different yields were observed between the three primer pools: Probe_10 produced the highest average yield (2.5 ± 0.87 µg) followed by Probe_20 (1.85 ± 0.81 µg) and Probe_28 (1.2 ± 1.0 µg) (Fig. 1b). Whole genome sequencing of the amplified products were used to compared the quality of genome coverage (number of bases with at least 5x coverage) by each set (pool) and no significant difference was found (Spearman’s correlation: Probe_10 and Probe_20, R^2^ = 0.97, *p* < 0.001; Probe_10 and Probe_28, R^2^ = 0.96, *p* < 0.001; Probe_20 and Probe_28, R^2^ = 0.97, *p* < 0.001). Probe_10 (Additional file 1: Table S2) was therefore chosen for all subsequent sWGA reactions based on amplification yield and cost.

### Coverage profile of sWGA samples

**Fig. 2:**
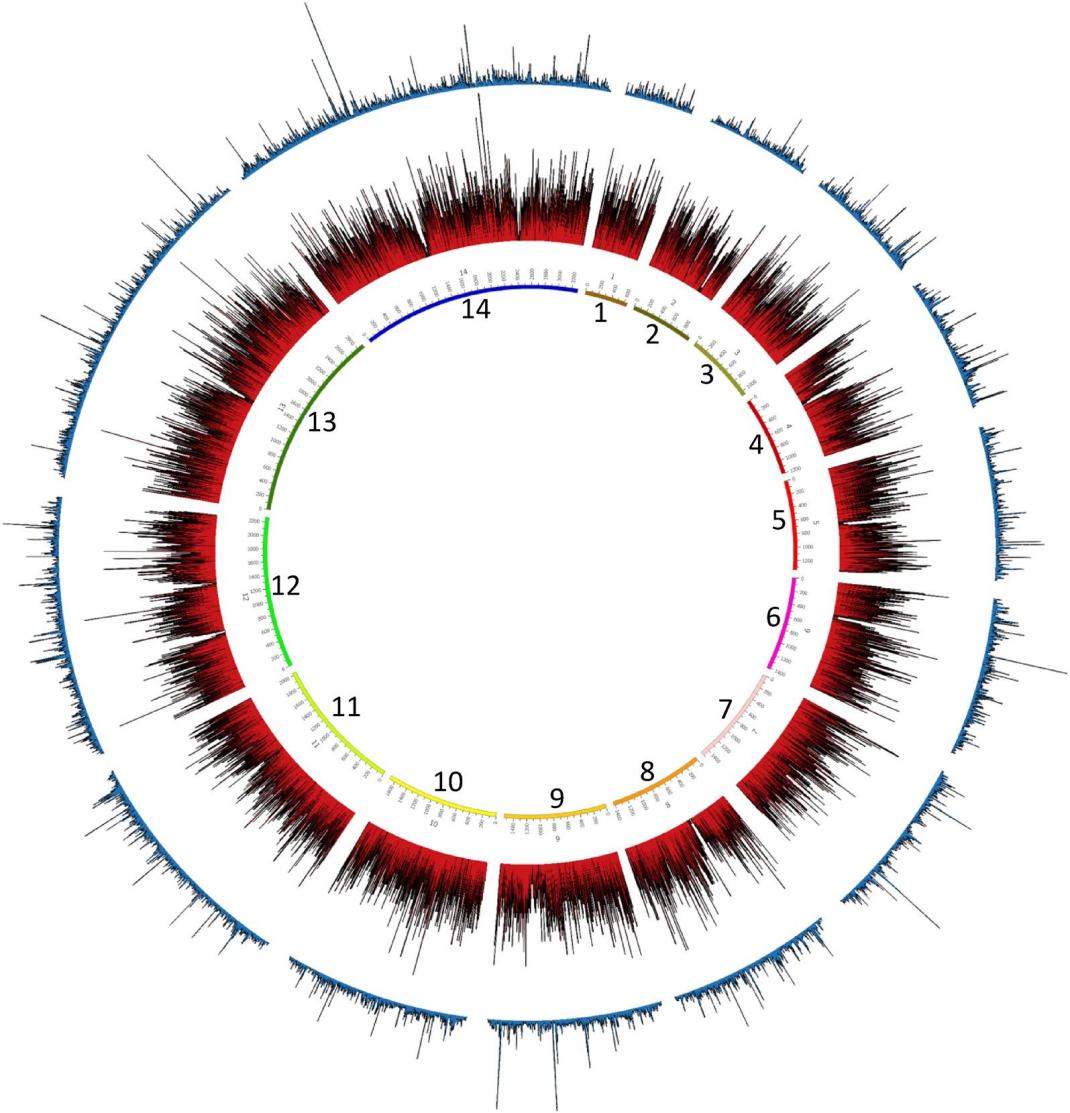
Circos plot analysis of Probe_10 primer pool and *P. falciparum* genome coverage. The *three rings* represent, from innermost to outermost, the 14 *P. falciparum* chromosomes and position in kb, the total number of primers binding in 1 kb windows (*red lines*), and the average read depth in 1 kb windows (*blue lines*). The figure was generated using the Circos software [21]

To perform a more in-depth analysis on Probe_10, a mock sample containing a mixture of human (96%) and *P. falciparum* (4%) DNA was amplified and sequenced, as described in Methods. Using the Illumina short read sequence data obtained, the primer binding positions as well as the short-read sequence alignments was plotted against the reference genome using Circos software [21] for data visualization. Figure 2 shows the probe binding sites (middle circle) as well as the sequence reads coverage profile (outermost circle) on all 14 chromosomes (inner circle) of the *P. falciparum* genome. Probe_10 successfully amplifies the majority of the parasite genome, but the variable subtelomeres are not adequately covered (Fig. 2; Additional file 1: Figure S1). However, the coverage profile is uneven and does not correlate properly with the primer binding sites (Pearson’s correlation R^2^ = −0.007, *p* = 0.3).

**Fig. 3:**
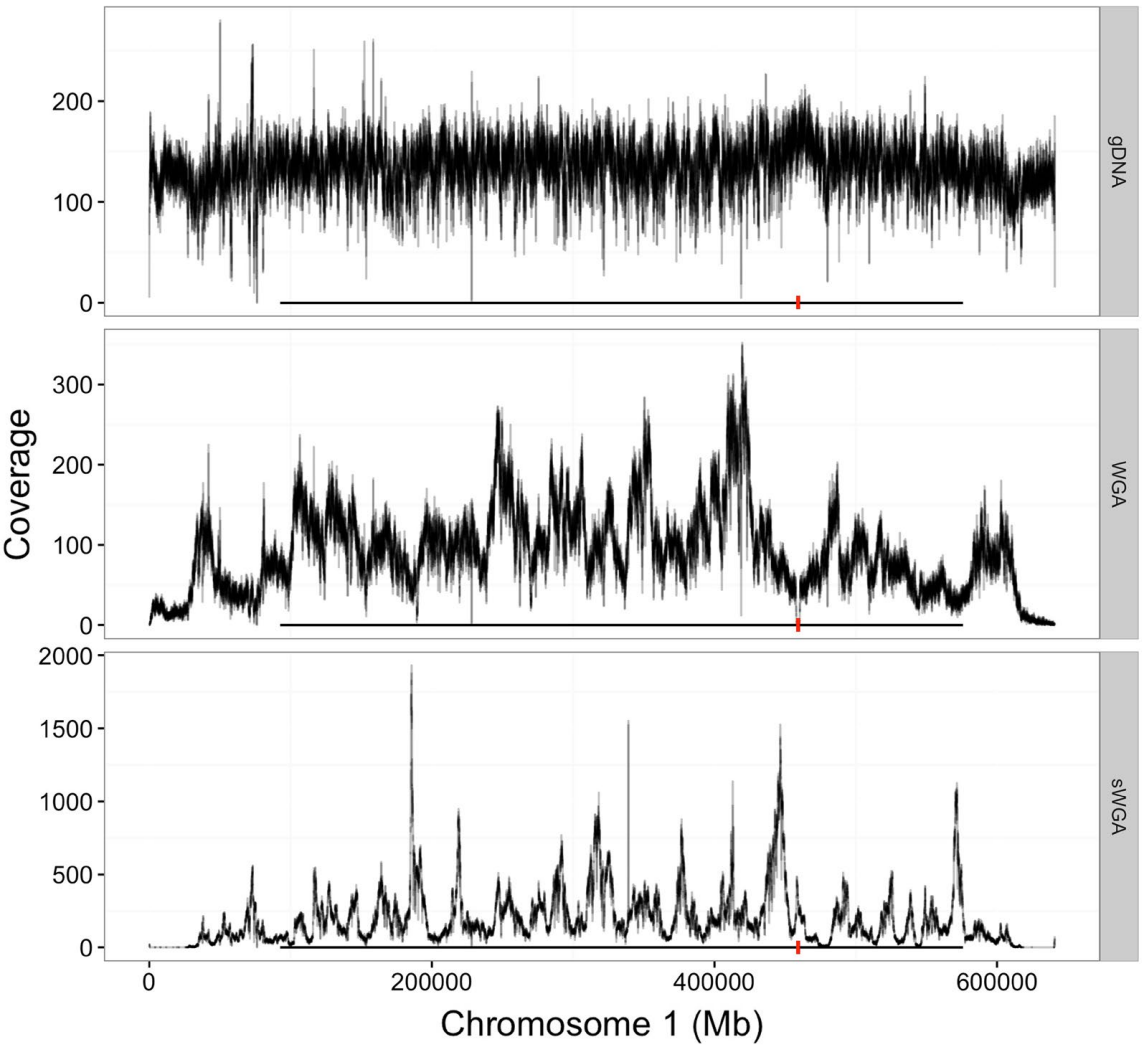
Core genome coverage profile. Coverage depth of chromosome 1 by leucodepleted and unamplified (VB), whole genome amplified (WGA) and selective whole genome amplified (sWGA) DNA of *P. falciparum* strain 3D7. *Black horizontal line* shows positions corresponding to the core genome and *red vertical line* shows the centromere

Previous analysis [15] revealed accessible and inaccessible regions in the *P. falciparum* genome. Inaccessible regions, mainly the telomeres, centromeres and sub-telomeres, are comprised of hypervariable and/or highly repetitive sequences that are difficult to assemble or map. The remaining parts (core genome) consist of mainly the coding sequences of relatively balanced-base composition, and are generally accessible in most genome analysis. In order to test whether sequences generated from sWGA samples would successfully cover the core genome, coverage profile of *P. falciparum* strain 3D7 samples sequenced as gDNA (3 samples without amplification), WGA DNA (3 samples amplified using optimized whole amplification method [12]) or sWGA (3 samples consisting of a mixture of 4% parasite and 96% human DNA, amplified using selective whole genome amplification method) was plotted. Figure 3 shows the coverage profile of the samples on chromosome 1, highlighting regions corresponding to the core genome. Unamplified DNA (gDNA) provided the most even and uniform coverage across the entire genome. Both WGA and sWGA samples produced relatively spiky and uneven coverage, with the sWGA producing higher coverage depths of uneven distribution.

### sWGA enriches for *Plasmodium falciparum* sequence reads

**Fig. 4:**
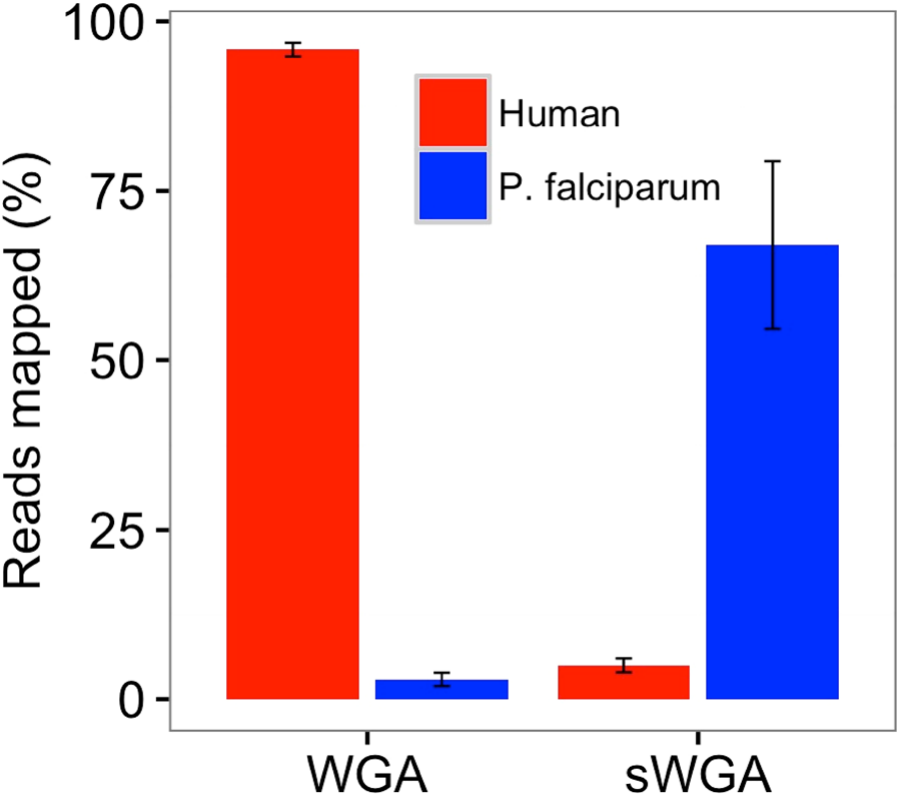
Selective whole genome amplification (sWGA) enrichment. Simulated clinical samples comprising 96% human DNA and 4% *P. falciparum* DNA (3D7) were amplified using either WGA or sWGA. Amplified samples were sequenced to determine the proportion of reads mapping to human or *P. falciparum* reference genomes

The sequence data was analysed by comparing datasets from standard whole genome amplified (WGA) samples against their selectively amplified (sWGA) counterparts to determine the level of enrichment. An average of 73.2% (sd 4.4; N = 5) of the reads in the sWGA-treated samples mapped to *P. falciparum*. More than 18-fold enrichment of parasite DNA was achieved, depending on the extent of host contamination in the original sample (Table 1; Fig. 4). In contrast, data obtained from DBS extracts and amplified by standard WGA (no selective amplification) had <1% of reads mapping to *P. falciparum* and the rest (>99%) mapping to the host genome (Table 1; Fig. 4), demonstrating the efficacy of sWGA in selective amplification of parasite DNA.

**Table 1:** sWGA enrichment analysis

	Reads mapping to:			
Sample	*P. falciparum* (%)	Human (%)	Others (%)	Fold enrichment
WGA_3D7_1	3.30	96.20	0.50	N/A
WGA_field_1	2.90	95.83	1.27	N/A
sWGA_3D7_1	79.74	3.33	16.94	19.93
sWGA_3D7_2	74.64	4.82	20.54	18.66
sWGA_Field_1	73.50	4.45	22.04	N/A
sWGA_Field_2	69.26	4.81	25.93	N/A
sWGA_Field_3	68.84	5.51	25.61	N/A

Mock and field samples were amplified by either WGA or sWGA before sequencing. Proportions of reads mapping to either human or *P. falciparum* genomes were used to determine the level of parasite DNA enrichment by sWGA treatment. 3D7 represent mock samples prepared by mixing P. falciparum and human genomic DNA in the ratio of 1:24 (4% parasite and 96% human). Field represent clinical genomic DNA samples extracted from dried blood spot filter papers

### Parasitaemia and genome coverage threshold in mock samples

**Fig. 5:**
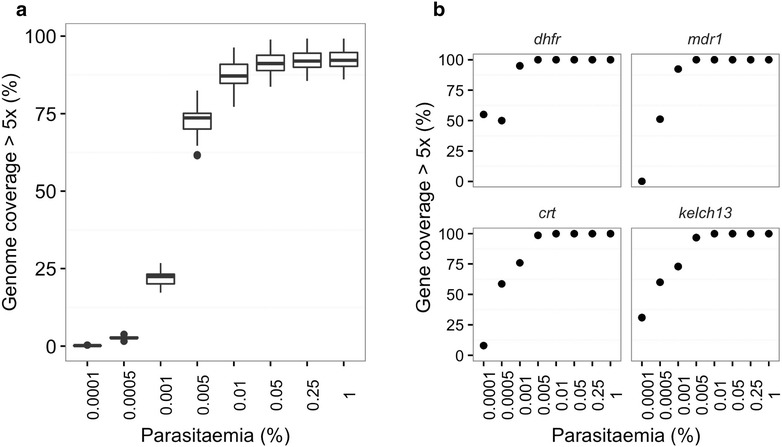
Assessing sWGA sensitivity and parasitaemia threshold. Clinical mock samples represent different levels of parasitaemia ranging from 0.0001 to 1%. Data from sWGA-processed samples were analysed to determine coverage of *P. falciparum* genome. **a** Genome coverage by samples of different parasitaemia levels. **b** Coverage of important drug resistance loci by mock samples of different levels of parasitaemia

To investigate the sensitivity of the sWGA application, genome coverage threshold by sequence data generated from samples with different levels of parasitaemia was analysed. In vitro infected red blood cells were mixed with human whole blood to simulate different levels of clinical parasitaemia ranging from 1.0 to 0.0001%. For samples with a parasitaemia of ≥0.005% [~6.25 parasites per 200 white blood cells (WBC)], ≥70% of the core nuclear 3D7 genome was covered at depth of ≥5× reads. However, the coverage dropped sharply for samples with parasitaemia below 0.005% (Fig. 5a; see Additional file 1: Figure S2 for detailed coverage distribution). The same dataset was used to analyse coverage of known important drug resistant loci in the genome [22]. As shown in Fig. 5b, a similar coverage profile was observed where all the 7 specified drug resistant loci were covered 100% at depths of ≥5× reads for samples with parasitaemia ≥0.005%.

### sWGA allows whole genome sequencing directly from clinical dried blood spots

Having established sWGA efficacy in mock blood samples, DBS field isolates collected from two sites in Ghana, with a parasitaemia ranging from 0.001 to 8.9% (1.25–11,125 parasites per 200 WBC or 40–356,000 parasites per µl of blood) were used to test the method. DNA was extracted from 205 DBS samples (average yield 116 ng, SD 116.7), which were subsequently subjected to sWGA (average yield 1399 ng, SD 502). From those, 156 (76%) passed the threshold of 500 ng for library preparation and were, therefore, whole genome sequenced.

**Fig. 6:**
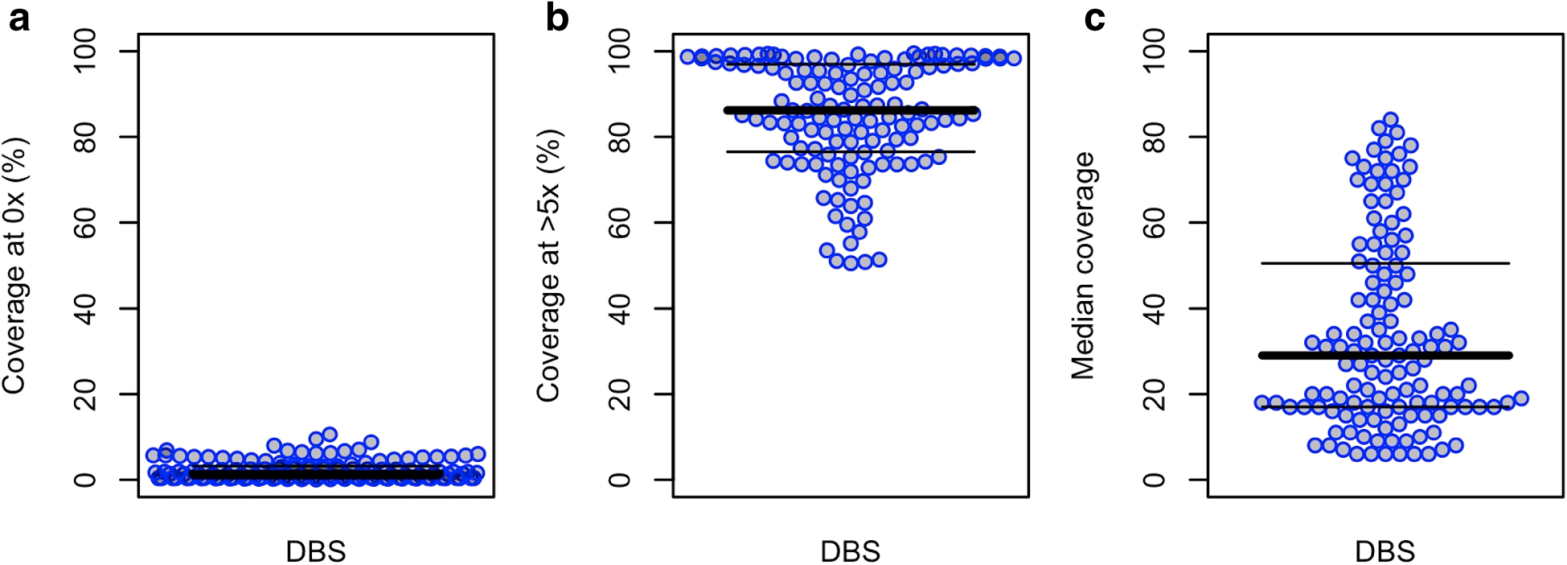
Core genome coverage of 156 dried-blood spot (DBS) clinical samples subjected to sWGA. **a** Percentage of genome positions with no coverage. **b** Percentage of genome positions with at least 5× coverage. **c** Median genome coverage

A total of 156 DBS samples were analysed, excluding those with <50% of the core genome covered at 5× reads or less (N = 25). On average only 2.3% (SD 2.3) of the core genome of the 131 DBS samples was not covered at all (Fig. 6a), whereas 85% (SD 13) of the core genome was covered at 5× or more (Fig. 6b). The median coverage of the core genome was 29× (Fig. 6c).

**Fig. 7:**
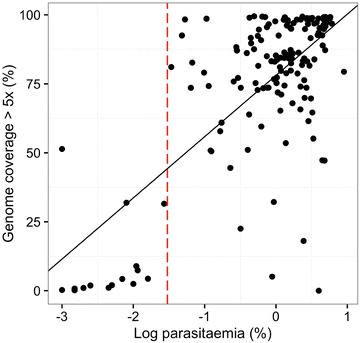
Field DBS samples with higher parasitaemia have a higher proportion of genome positions covered at depth >5×. (*F*_(1,150)_ = 135.5; p = 0.001). The parasitaemia threshold to obtain at least 50% of the genome covered at 5× is 0.03% (*dotted red line*)

As expected, samples with higher parasitaemia (above 0.1%) produced sequence data with better coverage at depths of ≥5×, whereas samples with parasitaemia lower than 0.03% had many positions covered at depths <5× (Fig. 7, *F*_(1,150)_ = 135.5, *p* < 0.001). In this dataset all samples with parasitaemia lower than 0.03% (N = 25) had more than 50% of the genome covered at 5× or less. There was one exception; a sample with 0.001% parasitaemia had 51.4% of the core genome covered at 5×. The samples with low parasitaemia had a much larger proportion of missing bases in the core genome (Fig. 7). Coverage of genes that are either responsible for, or associated with, anti-malarial drug resistance (Additional file 1: Figure S3) were analysed, and a general tendency of better coverage in samples of higher parasitaemia (>0.02%) was observed, while those with parasitaemia lower than 0.01% showed poor coverage across the genes.

Taken together, our data establishes 0.03% parasitaemia (40 parasites per 200 WBC) as the minimum threshold on which sWGA technology is capable of generating quality sequence data with coverage suitable for most genetic analyses on DBS field samples (Fig. 7, vertical dotted line marks the 0.03% parasitaemia threshold). The data also show that at least 180 parasite genomes per sample is required for efficient sWGA processing.

### High concordance between dried blood spot samples and venous blood samples

**Fig. 8:**
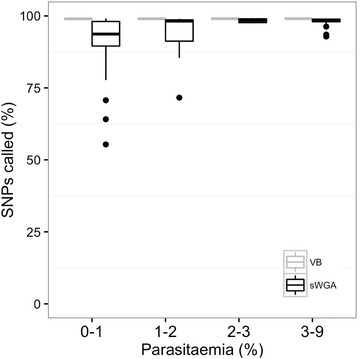
Single nucleotide polymorphism (SNP) calls from matching venous blood samples (VB) and dried blood spots samples (DBS). The percentage of SNPs called in DBS samples decreases as parasitaemia decreases

In order to further evaluate sWGA efficiency and suitability for genetic studies from DBS samples, a concordance analysis was performed using the set of 120 field samples with matched pairs of VB and DBS filter papers. Sequence data from both VB and DBS samples were analysed in parallel and genotyped against the ~1.2 million high quality SNP positions previously identified in the *P. falciparum* genome [15]. Genotype calls from matching VB (gDNA) and DBS (sWGA) sample pairs were analysed. In the gold standard VB samples, a median of more than 98% (N = 1,217,003) of SNPs were called in all the samples (Fig. 8). Overall, 93% (N = 1,154,911) of SNPs were called in the DBS samples, with a slight reduction at a lower parasitaemia (Fig. 8). The accuracy of the SNPs called was investigated by performing a concordance analysis between SNP calls made from VB and DBS samples. Samples that had <50% of the genome covered at 5× (N = 7) as well as all missing calls of the remaining DBS samples (total samples analysed N = 113) were excluded from this analysis. There was high concordance between the SNPs called in both VB and DBS samples, with an average of more than 99.9% (out of 1,241,840 SNPs, SD 10.97%) of calls being concordant (either Ref/Ref or Alt/Alt; Table 2). Only 0.04% (out of 1,241,840 SNPs, SD 0.08%) of calls were discordant (Ref/Alt or Alt/Ref).

**Table 2:** SNP concordance analysis of dried blood spots (DBS; sWGA) and venous blood (VB; leucodepleted and unamplified) samples (N = 113)

	Ref/Ref	Alt/Alt	Ref/Alt	Alt/Ref
Average	1,130,936.8 (99.82%)	1,391.3 (0.12%)	288.4 (0.02%)	283.4 (0.02%)
SD	124,553.8 (10.9%)	848.6 (0.07%)	477.8 (0.04%)	498.3 (0.04%)
Median	1,179,088 (99.9%)	1,026 (0.08%)	0	0

Genotype calls across ~1.2 million biallelic typable SNPs from matching VB and DBS (sWGA) sample pairs were analysed to obtain SNP concordance between the two sample processing methods. Ref, reference genotype call; Alt, alternative genotype call; Het, heterogeneous calls; Miss, missing calls

**Fig. 9:**
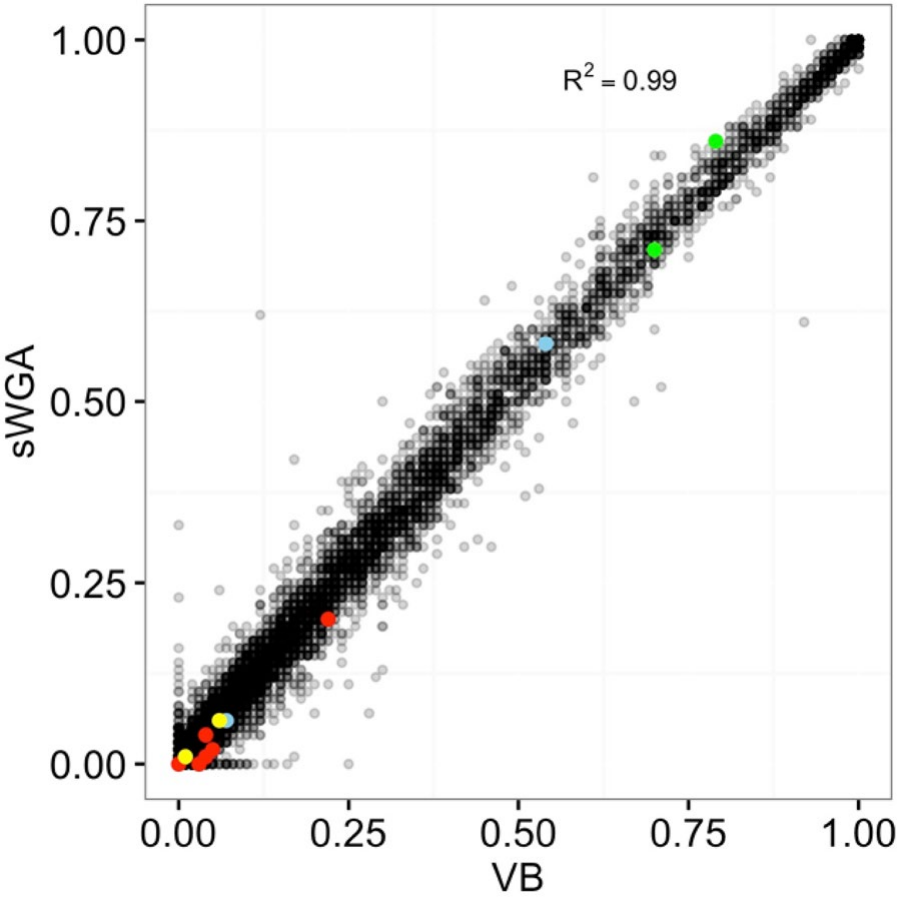
Population-level allele frequency of venous blood (VB) and DBS (sWGA) samples are strongly correlated. Using variant calls from the 1.2 million high quality-biallelic SNP positons, non-reference allele frequencies (NRAF) were compared from matching VB and DBS samples, and a strong correlation between the two sample sets was obtained. *Coloured dots* represent specific mutations of drug resistance genes. *Green dots* represent *dhfr* (N51I, C59R, S108N); *blue dots* represent *mdr1* (N86Y, Y184F); *red dots* represent *crt* (M74I, N75D, N75K, K76T, A220S, Q271E, I356T, R371I); *yellow dots* represent *dhps* (S436A, G437A, K540E, A581G, A613S)

The accuracy of SNP calls from sWGA-generated data was further tested using allele frequency concordance metrics. Using the VCF files targeting the 1.2 million high quality-biallelic SNPs, the population-level allele frequencies was analysed from matching VB and DBS samples, and strong correlation of non-reference allele frequencies (NRAF) was found between VB (gDNA) and DBS (sWGA) samples (Fig. 9; Pearson’s correlation *Ρ* = 0.99, *p* < 0.001). For more detailed analysis, allele frequencies of specific mutations for key malaria drug resistance genes—*dhfr*, *mdr1*, *crt*, *dhps* and *kelch13*—were analysed. Once again, high concordance between VB and DBS samples was observed (Fig. 9; Additional file 1: Table S1). In summary, after excluding lower quality samples with missing calls, very high concordance in population genetic data between VB and DBS samples was observed.

## Discussion

Using sWGA to amplify parasite DNA from dried blood spots has immediate and important implications for public health. This work has comprehensively evaluated the potential of sWGA method. Collecting clinical malaria samples as DBS on filter paper is field-friendly and has several advantages for both patient and researcher over the venous blood (VB) draw methods currently used for parasite whole genome sequencing [23]. Finger-prick sampling requires less advanced training than VB draws, collects ~50× less blood, and is more convenient for most patient groups. Unlike VB draws, DBS samples also do not require special facilities for transportation, refrigeration, and storage, since the blotted paper is stabilized by the membrane that preserves genetic integrity [23, 24]. VB sampling is thus relatively limited in geographic range, restricted to locations with well-established and resourced clinics. Sequencing from DBS samples would break this technical bottleneck, allowing significant expansion of sample collection to include very remote regions, increasing sampling density and coverage [23, 24].

The data presented show that ~110 ng of genomic DNA can be extracted from a 20–40 µl DBS, of which over 98% is host material. Isolating sequenceable parasite DNA from a DBS sample that is highly contaminated with host DNA has hindered applications of genetic tools in malaria research and control programmes. Previous studies have identified various methods to overcome the challenges of host DNA contamination in pathogen sequencing [4, 25]. However, most of these techniques require relatively large quantities of starting DNA material that is impossible to obtain from DBS samples. The approach described here provides a timely solution to this challenge, creating opportunities for both large-scale field isolate sequencing studies and analysing archived clinical samples that would otherwise be too contaminated and low yield for whole genome sequencing.

To thoroughly evaluate the quality and accuracy of sWGA sequence data for genetic analysis of clinical malaria samples, 156 DBS collected from clinical malaria patients was sequenced and analysed using sWGA. 120 of these had their corresponding VB counterparts collected simultaneously, allowing direct comparison between DBS (sWGA) and VB (leucodepleted and unamplified) WGS data from an identical patient cohort [5, 6]. More than 75% of the *P. falciparum* genome was covered at ≥5× in 117 (97.5%) DBS samples for which parasitaemia was ≥0.03%. The sWGA-derived genome sequences show a less uniform coverage profile compared to data generated from unamplified genomic DNA (VB-derived, Fig. 3). This is typical of whole genome amplified data [12]. However, the core genome was adequately covered at depths suitable for most downstream analysis including variant detection and SNP genotyping. Further optimization is required to amplify and successfully genotype regions outside the core genome, such as telomeres and mitochondria.

The high concordance of SNP calls and allele frequencies between the DBS and VB paired samples indicates that samples that were subjected to sWGA are suitable for population genetic studies. Significantly for the potential applicability of this technology to public health surveillance projects, important malaria drug resistance loci were successfully sequenced and showed very similar allele frequencies for both DBS and VB samples (Fig. 9; Additional file 1: Table S1).

## Conclusion

In summary, this work shows that processing DBS samples using sWGA method produces reliable sequence data, provided that: the sample has ≥180 *P. falciparum* genomes (parasitaemia threshold ~0.03%, or ~40 parasites per 200 WBC); the threshold for library preparation is met (≥500 ng of DNA post-sWGA); and the sequence data obtained covers at least 50% of the genome at a depth of 5× or more. Samples with much a lower parasitaemia, for example those collected from asymptomatic patients or during the low transmission season, will require further optimization to improve sensitivity and coverage. Using sWGA technology, genomic data from larger sample sizes with geospatial resolution could provide useful information to public health bodies, for example through rapid detection of emerging patterns in parasite evolution in response to control initiatives such as anti-malarial drugs.

## Additional files

**Additional file 1: Figure S1.** A plot of primers (probes) and their binding distribution on the *P. falciparum* genome. The topmost panel show cumulating binding positions and distribution profile of all the 28 primers. Black dots (1) show positions where the primer binds and Red (0) dots shows positions with no primer binding. Probes are ordered from bottom to top; the first 10 primers is Probe_10, followed by Probe_20 then Probe_28. **Figure S2.** Coverage depths frequencies of samples with different parasitaemia levels. **Figure S3.** Coverage of genes associated with drug resistance. Colours reflect the percentage of genome covered, ranging from 5x (grey) to 30x or more (red). **Table S1.** Non-reference allele frequencies (NRAF) of major drug resistance genes for venous blood (VB; leucodepleted and unamplified) and dried blood spots (DBS; sWGA) samples. Gene name, chromosome number, position, mutation name, mutation type and the NRAF found in West Africa populations (MalariaGen https://www.malariagen.net/apps/pf/4.0/) are shown. Notably, the studied populations have high *dhfr* mutation frequencies and rare *crt* mutations. Presumably because the use of SP was widespread and is still being used (e.g. pregnancy prophylaxis), whereas enough time has passed since chloroquine was widely used [26–28]. **Table S2.** Probe_10. sWGA primers for *Plasmodium falciparum*.
