# Supplementary material for: Whole genome sequencing of Plasmodium falciparum from dried blood spots using selective whole genome amplification
Source: Malar J. 2016 Dec 20;15:597. doi: 10.1186/s12936-016-1641-7 (PMC5175302; doi:10.1186/s12936-016-1641-7)
Supplement: Supplementary file 1 — Additional file 1: Figure S1. A plot of primers (probes) and their binding distribution on the P. falciparum genome. The topmost panel show cumulating binding positions and distribution profile of all the 28 primers. Black dots (1) show positions where the primer binds and Red (0) dots shows positions with no primer binding. Probes are ordered from bottom to top; the first 10 primers is Probe_10, followed by Probe_20 then Probe_28. Figure S2. Coverage depths frequencies of samples with different parasitaemia levels. Figure S3. Coverage of genes associated with drug resistance. Colours reflect the percentage of genome covered, ranging from 5x (grey) to 30x or more (red). Table S1. Non-reference allele frequencies (NRAF) of major drug resistance genes for venous blood (VB; leucodepleted and unamplified) and dried blood spots (DBS; sWGA) samples. Gene name, chromosome number, position, mutation name, mutation type and the NRAF found in West Africa populations (MalariaGen https://www.malariagen.net/apps/pf/4.0/) are shown. Notably, the studied populations have high dhfr mutation frequencies and rare crt mutations. Presumably because the use of SP was widespread and is still being used (e.g. pregnancy prophylaxis), whereas enough time has passed since chloroquine was widely used [26–28]. Table S2. Probe_10. sWGA primers for Plasmodium falciparum. [file 12936_2016_1641_MOESM1_ESM.pptx]

## Slide 1
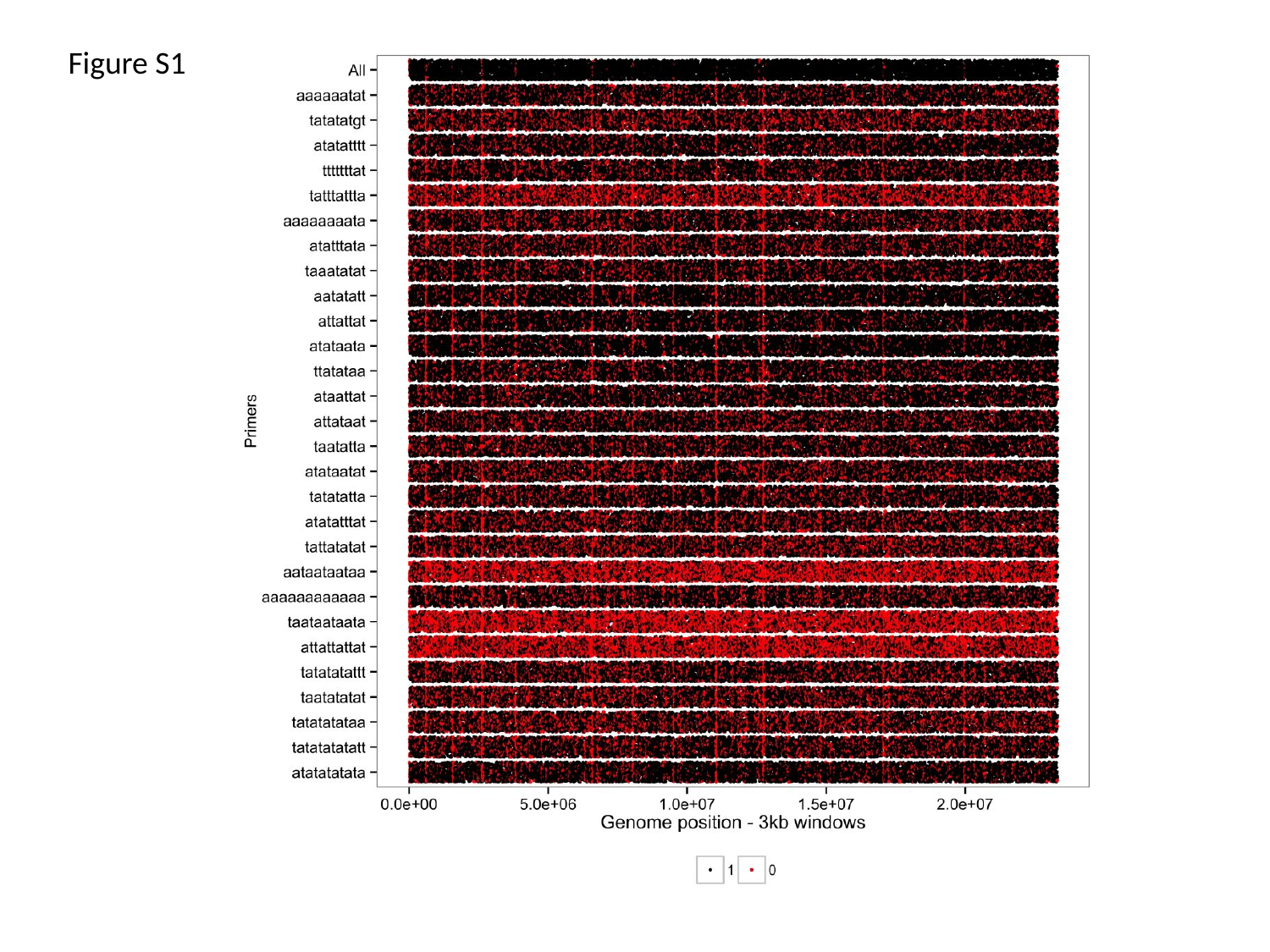

Figure S1

## Slide 2
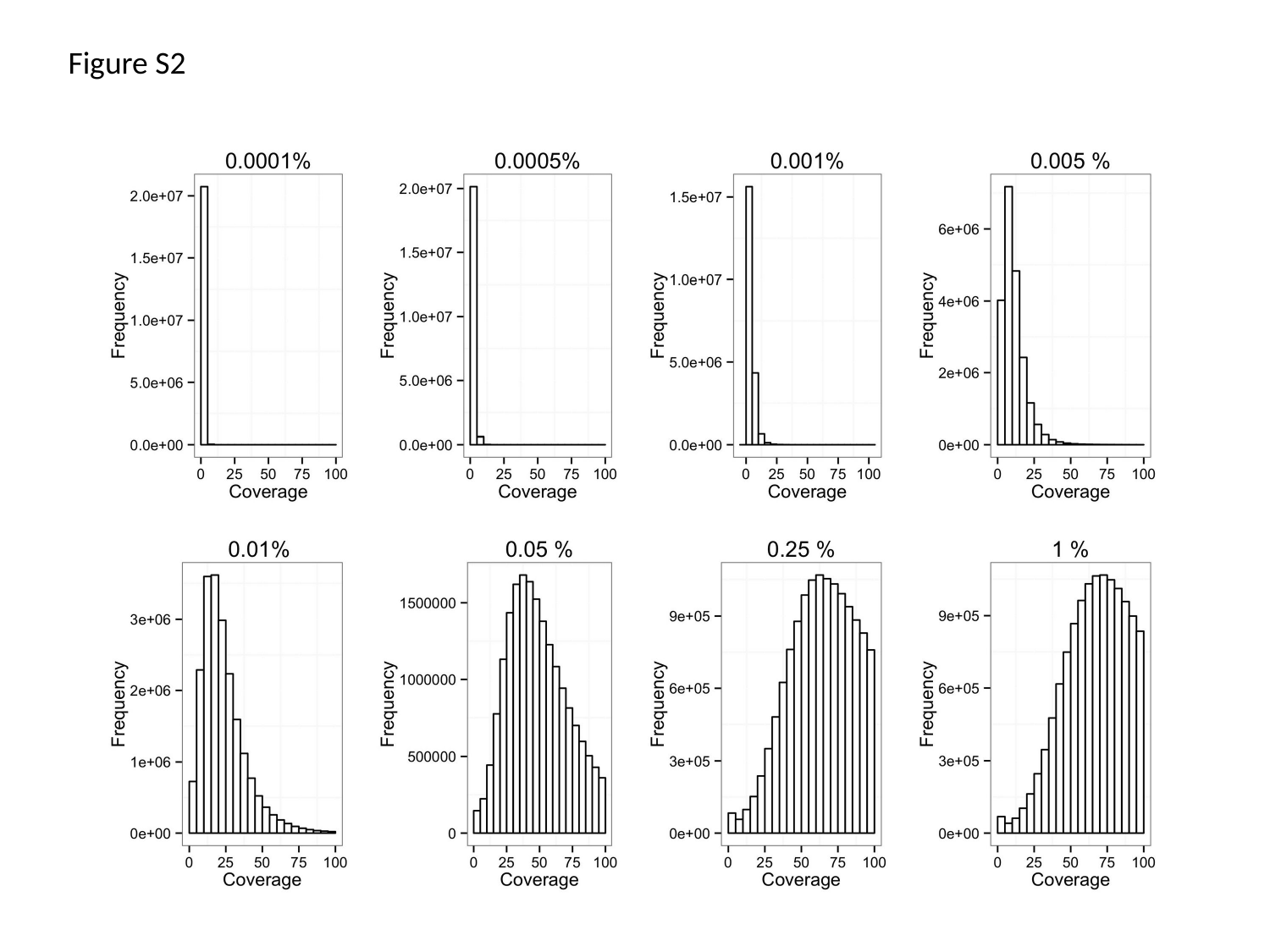

Figure S2

## Slide 3
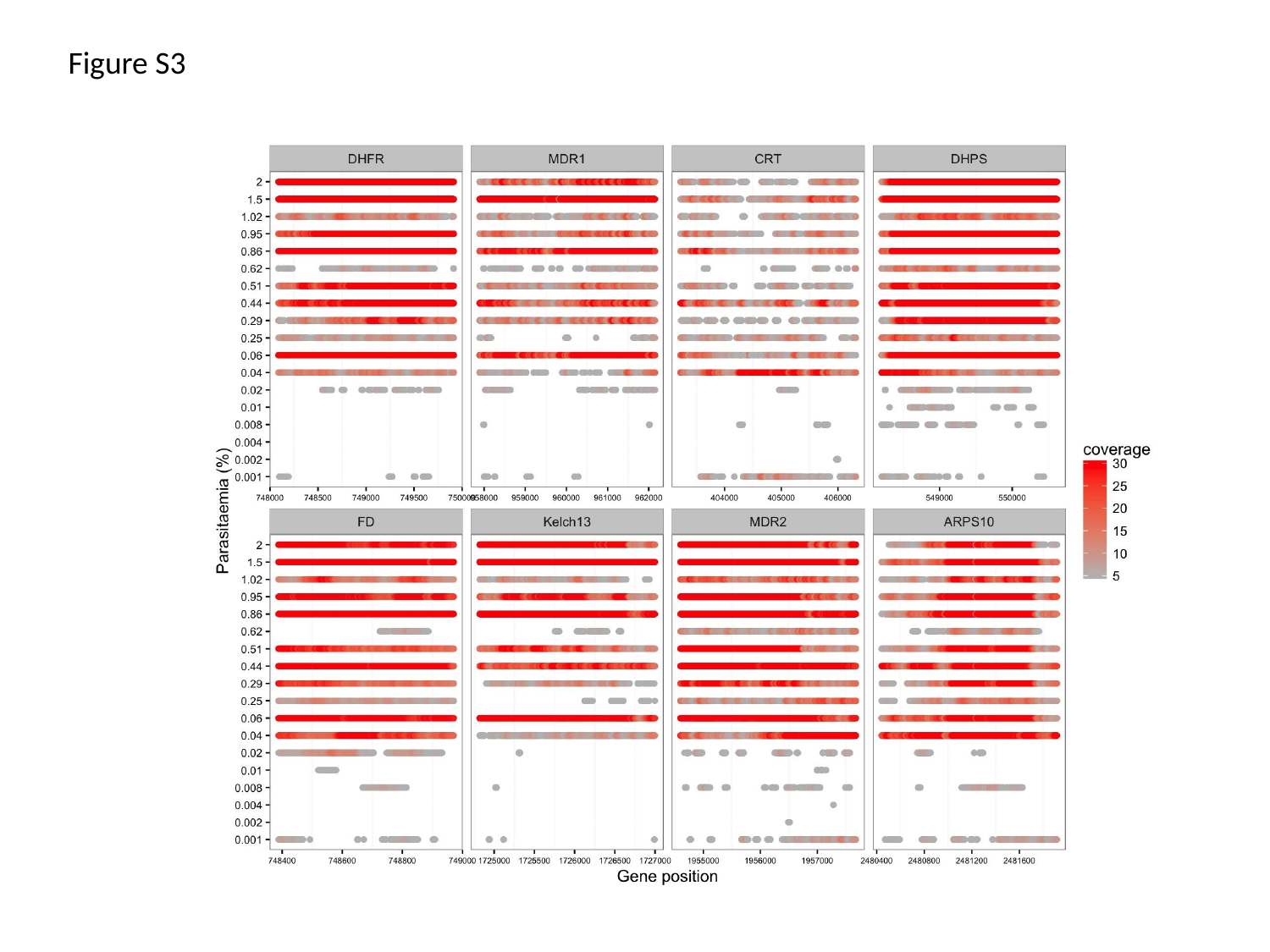

Figure S3

## Slide 4
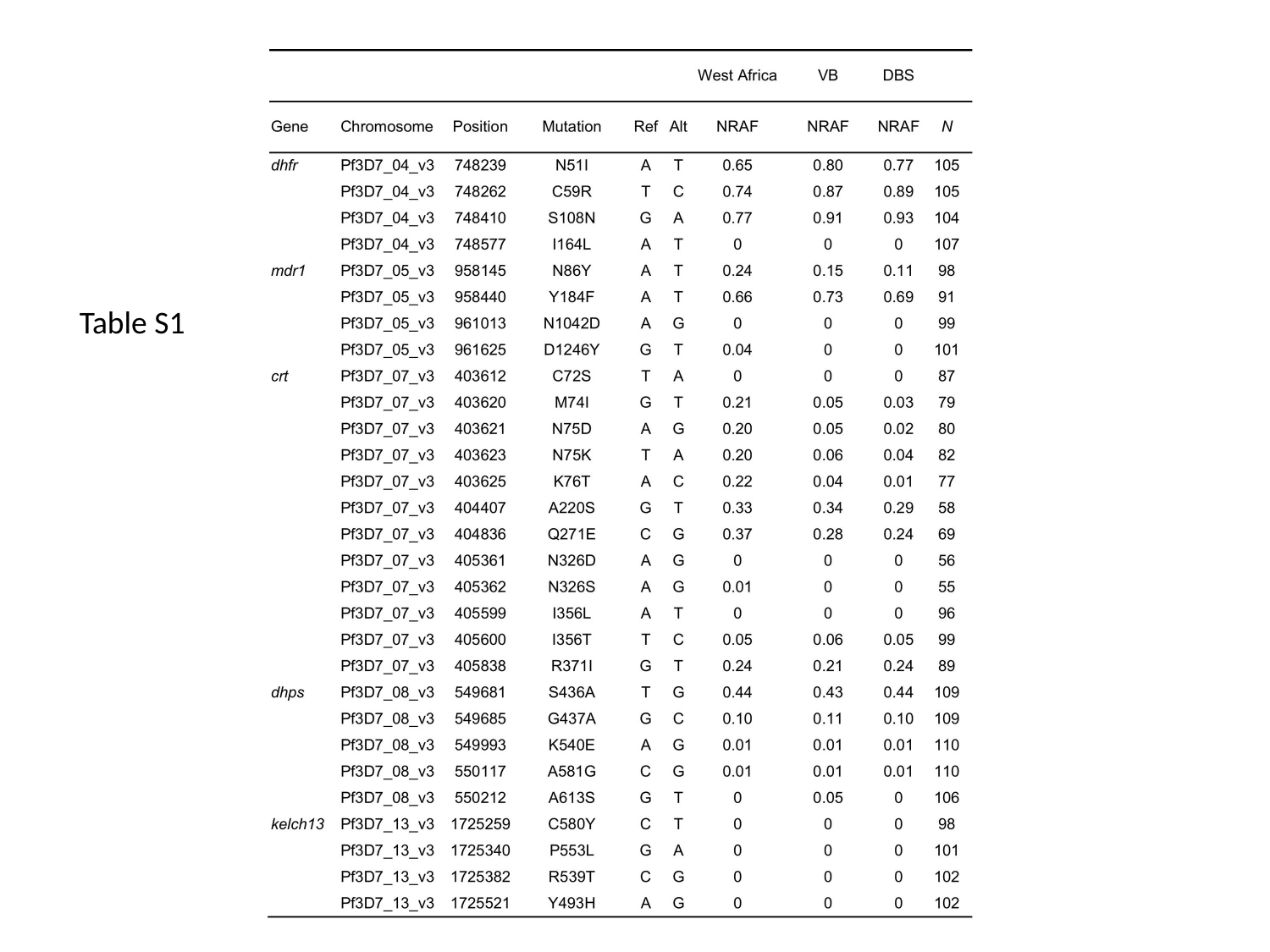

Table S1

## Slide 5
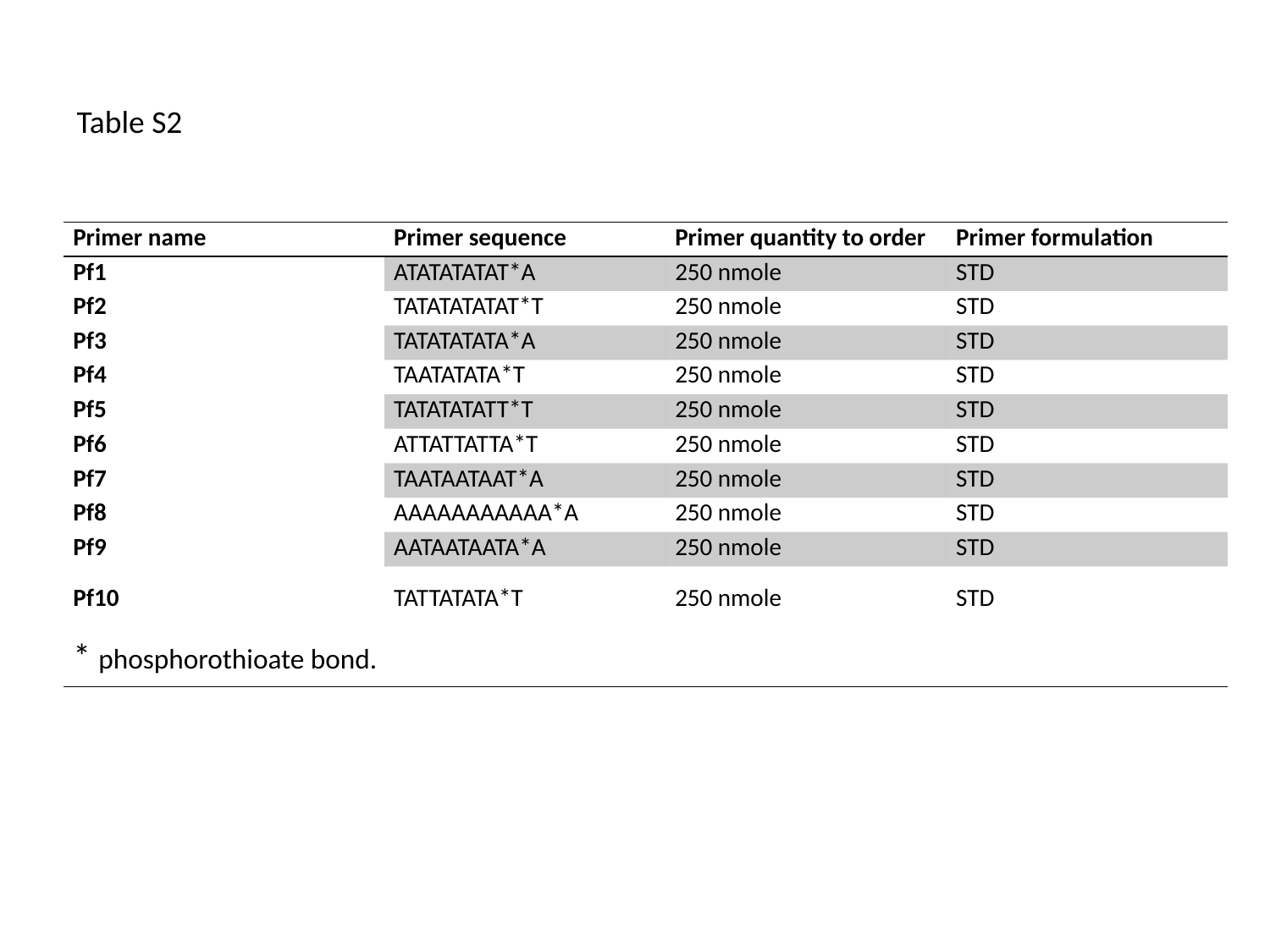

Table S2
| Primer name | Primer sequence | Primer quantity to order | Primer formulation |
| --- | --- | --- | --- |
| Pf1 | ATATATATAT\*A | 250 nmole | STD |
| Pf2 | TATATATATAT\*T | 250 nmole | STD |
| Pf3 | TATATATATA\*A | 250 nmole | STD |
| Pf4 | TAATATATA\*T | 250 nmole | STD |
| Pf5 | TATATATATT\*T | 250 nmole | STD |
| Pf6 | ATTATTATTA\*T | 250 nmole | STD |
| Pf7 | TAATAATAAT\*A | 250 nmole | STD |
| Pf8 | AAAAAAAAAAA\*A | 250 nmole | STD |
| Pf9 | AATAATAATA\*A | 250 nmole | STD |
| Pf10 | TATTATATA\*T | 250 nmole | STD |
| \* phosphorothioate bond. | | | |
